# Sulbactam–Durlobactam: A Novel Antibiotic Combination for the Treatment of *Acinetobacter baumannii*–Calcoaceticus Complex (ABC) Hospital-Acquired Bacterial Pneumonia and Ventilator-Associated Bacterial Pneumonia

**DOI:** 10.1155/cjid/2001136

**Published:** 2025-01-29

**Authors:** Wissam K. Kabbara, Elina Sadek, Hanine Mansour

**Affiliations:** , Department of Pharmacy Practice, School of Pharmacy, Lebanese American University, Chaghoury Building, 6 Floor, P.O. Box 36/ S 32, Byblos, Lebanon

**Keywords:** *Acinetobacter baumannii*–calcoaceticus complex (ABC), B-lactamase inhibitors, carbapenem-resistant *Acinetobacter* baumannii (CRAB), HAP, sulbactam–durlobactam, VABP

## Abstract

**Purpose:** To evaluate the pharmacology, pharmacokinetics, pharmacodynamics, antimicrobial activity, efficacy, safety, and the regulatory status of sulbactam–durlobactam.

**Summary:** Sulbactam–durlobactam is a recently approved antimicrobial combination of two *β*-lactamase inhibitors for the treatment of hospital-acquired bacterial pneumonia (HABP) and ventilator-associated bacterial pneumonia (VABP) associated with *Acinetobacter baumannii*–calcoaceticus complex (ABC) in patients 18 years and older. Sulbactam is a direct antibacterial activity with high susceptibility to *A. baumannii* species. Durlobactam, diazabicyclooctane *β*-lactamase inhibitors possesses a broad-spectrum activity against Ambler class A, C, and D serine *β*-lactamases. This combination has been studied to overcome resistance to ABC species. Data were obtained from in vitro and preclinical studies as well as phase I, II, and III clinical studies published in English between 200t0 and January 2023. A phase II trial showed similar tolerability and pharmacokinetics parameters of sulbactam–durlobactam in patients with urinary tract infections to placebo. However, no ABC infections were included in the trial. ATTACK, a phase III clinical trial of sulbactam–durlobactam, studied the safety and efficacy of sulbactam–durlobactam in patients with ABC HABP/VABP and showed its noninferiority to colistin. Reported adverse events include anemia, elevated liver enzymes, hyperkalemia, headache, diarrhea, nausea, urticaria, and vascular pain. Sulbactam–durlobactam is a new *β*-lactamase inhibitors combination active against MDR Gram-negative bacteria including ABC. It is currently approved for the treatment of HABP/VABP caused by susceptible ABC strains.

**Summary**

• Sulbactam–durlobactam is a new *β*-lactamase inhibitors combination with activity against multidrug-resistant *Acinetobacter baumannii*–calcoaceticus complex (ABC).
• It is indicated in patients 18 years of age and older who have limited or no alternative treatment options, for the treatment of hospital-acquired bacterial pneumonia and ventilator-associated bacterial pneumonia caused by strains of ABC.
• Most common adverse effects include liver test abnormalities, anemia, hypokalemia, headache, diarrhea, nausea, and vascular pain.

## 1. Introduction

Hospital-acquired pneumonia (HAP) or nosocomial pneumonia, the most common cause of hospital-acquired infection in the United States and Europe, is defined as a lung infection occurring in 48 h or more after hospital admission. Ventilator-associated bacterial pneumonia (VABP) is subclass of HAP. It occurs in 10%–20% of intensive care units' (ICU's) mechanically ventilated patients, at least 48–72 h after tracheal intubation [1–3]. Aerobic Gram-negative bacilli (e.g., *Escherichia coli, Klebsiella pneumoniae, Enterobacter* spp*, Acinetobacter* spp, and *Pseudomonas aeruginosa*) and Gram-positive cocci (e.g., *Streptococcus* spp [2] and *Staphylococcus aureus,* which includes methicillin-resistant *S. aureus*) are considered the most common causative microorganisms for HAP and VAP. In the United States, the common pathogens associated with VAP are *S. aureus* accounting for 20%–30% of isolates, P. aeruginosa accounting for 10%–20% of isolates, enteric Gram-negative bacilli accounting for 20%–40% of isolates, and lastly *A. baumannii* accounting for 5%–10% of isolates [4]. Also, these same microorganisms are mostly known in international surveillance programs, with a higher percentage of attributed cases to *P. aeruginosa* and *A. baumannii* [5] Additionally, many of these microorganisms are identified as multidrug-resistant bacteria. It is important to note that the prevalence of *Acinetobacter* species increased throughout the years as noted in several studies between 1994 and 2012 [5]. Furthermore, HAP infections caused by *A. baumannii* isolates are more common in Asia than the other continents, where 56%–61% of the isolates were resistant to carbapenems [5]. The use of antibiotics such as tigecycline and the polymyxins has been obstructed by their inadequate efficacy, safety concerns, pharmacokinetic profile, and rising rates of resistance [2]. The Infectious Diseases Society of America (IDSA) guidelines recommend either a carbapenem or ampicillin/sulbactam as a weak, low-quality recommendation for the treatment of HAP/VAP caused by susceptible *Acinetobacter* species and strongly recommend either one of the polymyxins. Additionally, they weakly recommend the use of inhaled polymyxins as an adjuvant therapy. On the other hand, the use of rifamycin as a combination or tigecycline is not recommended. Sulbactam is a first-generation *β*-lactam-*β*-lactamase inhibitor with direct activity against *A. baumannii* species and, in high doses, successfully reaches PBPs targets of *A. baumannii* isolates [6]. According to the guidelines, sulbactam continues to be considered the preferred empirical agent to treat suspected *A. baumannii* infections [7]. However, with the increase of *Acinetobacter* species resistance to sulbactam and carbapenems, several studies explored its combination with other antibiotics such as cefoperazone and tigecycline, polymyxins in addition to ampicillin [6, 8].

Such combinations, specifically with polymyxins, have led to a potential increase in toxicity namely nephrotoxicity without a strong outcome [5].

Durlobactam has a broad-spectrum activity against Ambler class A, C, and D serine *β*-lactamases and is considered a new member of the diazabicyclooctane class of *β*-lactamase inhibitors [9]. Therefore, with the emergence of resistant *Acinetobacter* species to the currently available treatment, the development of novel agents is crucial. Sulbactam–durlobactam (Xacduro) acquired The Food and Drug Administration (FDA) approval in April 2023 for the treatment of adults *Acinetobacter baumannii*–calcoaceticus complex (ABC), hospital-acquired bacterial pneumonia (HABP), and VABP [10]. This article evaluates the combination of sulbactam–durlobactam (Xacduro) in all aspects including its potential role in the treatment of HABP and VABP associated with ABC.

## 2. Data Selection

In vitro, phase I, II, and III clinical studies published in English between 2000 and January 2023 were reviewed in order to review the characteristics and the role of sulbactam–durlobactam including its chemical properties, structure, pharmacologic effect, and its safety and efficacy in the treatment of adult patients with HABP and VABP associated with ABC.

### 2.1. Chemistry and Pharmacology

**Figure 1:**
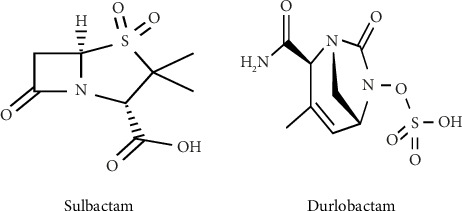
Chemical structure of sulbactam and durlobactam [9].

Sulbactam is a first-generation sulfone *β*-lactam-*β*-lactamase inhibitor with a chemical structure closely resembling penicillin (Figure 1). It is a semisynthetic penicillanic acid, usually coformulated with ampicillin, to treat *β*-lactamase-producing bacterial pathogens. The activity of sulbactam is limited to Ambler class A serine beta-lactamases [9]. Sulbactam also acts as a bactericidal cell wall synthesis inhibitor that blocks vital penicillin-binding proteins (PBPs) in some Gram negatives, including ABC [11]. A study has shown that when sulbactam ratio increases in combination with penicillins and cephalosporins, the in vitro antimicrobial activity against *Acinetobacter* species is enhanced. Sulbactam inhibits PBP1a, PBP1b, and PBP3, but not PBP2 in *Acinetobacter* species [12]. The in vitro susceptibility of *Acinetobacter* species to sulbactam has been decreasing due to its inactivation by numerous upregulated *β*-lactamases, such as Ambler class A TEM-1, class C, and several class D *β*-lactamases [13].

Durlobactam, which is previously called ETX2514, is a new-generation *β*-lactamase inhibitor with a broad spectrum of activity when compared to the currently available *β*-lactamase inhibitors (Figure 1) [14]. It is a non-*β*-lactam diazabicyclooctane compound. This novel cyclic boronate compound possesses activity against Ambler class A enzymes: CTX-M-, TEM-, PER-, and SHV-type extended spectrum beta-lactamases (ESBLs), KPC carbapenemase. It also has activity against class C (ADC-type) and against class D (OXA-type) enzymes [14, 15]. Its main advantage as compared to other *β*-lactamase inhibitors is its ability to inactivate class D carbapenemases of the OXA family, which are prevalent in *Acinetobacter* species [14]. Durlobactam is not active against class B metallo-*β*-lactamases (MBL) which are usually not common in *Acinetobacter* species. The *β*-lactamase enzyme is reversibly carbamylated on the active site serine nucleophile and the cyclic urea is opened upon exposure to durlobactam [16]. Some studies have also shown that durlobactam has inherent antibacterial activity against few bacterial species; however, durlobactam, as a single agent does not possess an antibacterial activity against ABC isolates [9, 15].

When combined with durlobactam, sulbactam forms a potent combination that enhances its activity against resistant bacterial microorganisms [16]. Durlobactam's inhibition of Ambler class A, C, and D *β*-lactamases allows sulbactam to reestablish its bactericidal activity on the resistant *Acinetobacter* strains [16]. This synergy allows successful bacterial cell wall synthesis inhibition.

### 2.2. Spectrum of Activity and Resistance

Sulbactam is a *β*-lactam and *β*-lactamase inhibitor that is often added to *β*-lactam antibiotics, such as ampicillin, to extend their spectrum of activity. It can help to overcome resistance mechanisms and broaden the antibiotic's spectrum of activity. The specific spectrum of activity will depend on the particular *β*-lactam antibiotic added to sulbactam. The spectrum of activity of sulbactam when combined with a *β*-lactam antibiotic is primarily against bacteria that produce beta-lactamase enzymes. These bacteria include many strains of *Staphylococcus, Escherichia coli, Klebsiella, Haemophilus influenzae*, and anaerobes [17].

Though sulbactam by itself does not possess substantial antibacterial activity, it has shown modest in vitro activity against strains of *Acinetobacter baumannii* [18]. This is due to inhibiting vital enzymes needed for the synthesis of the bacterial peptidoglycan. It inhibits PBP1a, PBP1b, and PBP3, but does not inhibit PBP2.

Sulbactam's activity against *Acinetobacter* species is reestablished upon the addition of durlobactam.  Table 1 summarizes different studies that characterize in vitro activity of sulbactam–durlobactam against several isolates of ABC. For instance, one study showed that the sulbactam–durlobactam minimum inhibitory concentrations (MICs) MIC50 and MIC90 were 1 and 2 μg/mL, respectively, for all ABC isolates tested [20]. Adding durlobactam (at a fixed concentration of 4 μg/mL) to sulbactam reduced its MIC50 by 8 times (from 8 to 1 μg/mL) and its MIC90 by 32 times (from 64 to 2 μg/mL) for all ABC isolates. Sulbactam–durlobactam exhibited consistent in vitro effectiveness across various ABC species including MDR and extensively drug-resistant (XDR) isolates, when a preliminary breakpoint of ≤ 4 mg/L was used to define susceptibility. Sulbactam–durlobactam inhibited 98.3% of all ABC isolates and more than 96% of isolates resistant to sulbactam, imipenem, ciprofloxacin, colistin, amikacin, and minocycline. Sulbactam–durlobactam also inhibited 96.9% (2410/2488) of CRAB isolates. Only 84 out of 5032 isolates (1.7%) had MIC values greater than 4 μg/mL to sulbactam–durlobactam [20].

Another study examined the in vitro activity of sulbactam–durlobactam against 982 *A. baumannii* isolates [21]. The isolates were gathered from infections of the lower respiratory tract, abdominal tract, urinary tract, and skin and skin structures. The MIC50 and MIC90 of sulbactam–durlobactam for all A. baumannii isolates tested were 1 μg/mL and 4 μg/mL, respectively. Adding durlobactam to sulbactam reduced its MIC50 by 32 times (from 4 to 1 μg/mL) and its MIC90 by 16 times (from 64 to 4 μg/mL). Sulbactam–durlobactam showed better activity as compared to imipenem and tigecycline, and it retained its activity against the carbapenem-resistant isolates [21].

Also, the susceptibility of 246 different CRAB isolates to sulbactam–durlobactam, amikacin, colistin, minocycline, and sulbactam was tested using the broth microdilution method [21]. The isolates were collected from 37 countries across six world regions. Sulbactam–durlobactam showed enhanced activity as compared to amikacin, minocycline, and sulbactam. The MIC50 and MIC90 of sulbactam–durlobactam were 1 μg/mL and 4 μg/mL, respectively. Colistin was the sole antibiotic that showed similar activity to sulbactam–durlobactam [22].

Moreover, a study conducted in Greece included 190 *A. baumannii* isolates, which were collected from hospitalized patients. Sulbactam–durlobactam was compared to amikacin, minocycline, sulbactam, and colistin. The sulbactam–durlobactam MIC50 and MIC90 were 4 and 8 μg/mL, respectively. Durlobactam restored the activity of sulbactam against the majority of tested strains, and the combination of sulbactam–durlobactam had a superior activity as compared to colistin, minocycline, and amikacin [19].

Miller et al. detailed the analysis of the original ABC isolates obtained from patients participating in the ATTACK trial and included an investigation of how microbiological results correlate with sulbactam–durlobactam MIC values and the genetic factors influencing resistance to sulbactam/durlobactam [23]. The 175 ABC baseline isolates showed an MIC range of 0.25–32 μg/mL for sulbactam–durlobactam, with MIC50 and MIC90 values of 2 μg/mL and 4 μg/mL, respectively. The in vitro activity of sulbactam–durlobactam against resistant subsets of the ABC isolates showed an MIC range of 0.5–16 μg/mL to carbapenem-resistant, MDR, and XDR isolates. The MIC range to pan-drug-resistant strains was 1–8 μg/mL [23].

Favorable microbiological outcomes were seen across all sulbactam–durlobactam MIC categories, ranging from 0.5 to 4 μg/mL. Interestingly, eradication rates were not correlated with sulbactam–durlobactam MIC values up to 4 μg/mL [23]. Higher eradication rates were noted in patients with ABC isolates having a sulbactam–durlobactam MIC of 4 μg/mL compared to those with an MIC of 0.5 μg/mL, possibly due to a smaller number of patients with lower MIC levels. Favorable clinical and microbiological outcomes were seen in 4 patients with initially resistant ABC isolates (MIC = 8–16 μg/mL) who received sulbactam–durlobactam treatment, supporting the FDA-approved intermediate susceptibility breakpoint for sulbactam–durlobactam of 8 μg/mL [23].

The mechanisms of sulbactam resistance in ABC isolates involve the production of *β*-lactamases (including class A (TEM-1), class C (ADC-30), class D (OXA), and class B metallo-*β*-lactamases), alterations in PBPs, increased activity of efflux pumps, and the loss of outer membrane porins. Sulbactam–durlobactam is ineffective against ABC isolates that produce Ambler class B metallo-*β*-lactamases or have modifications in the active target site of sulbactam. Different types of beta-lactamases can be produced by the same ABC isolate and can exhibit variations in the amino acid sequences of the penicillin-binding proteins [24].

Current multidrug-resistant *A. baumannii* isolates exhibit variability in both the number and diversity of *β*-lactamase genes encoded by each individual strain. An analysis of 84 clinical genetically different clinical isolates from various regions demonstrated that every strain carried a minimum of 2 and up to 5 unique *β*-lactamase genes. More than 50% of the isolates also carry at least one gene encoding a multi–class A *β*-lactamase. For this collection of strains, the MIC90 of meropenem was ≥128 mg/L, while the MIC90 for sulbactam–durlobactam was 4 mg/L [14].

In vitro, rare spontaneous mutants exhibiting resistance to sulbactam were identified, and their genetic changes were found to be located in PBP3 active site. *Acinetobacter baumannii* strains carrying the S390T and S395F mutations in PBP3 displayed significantly slower growth rates. This suggests that *Acinetobacter baumannii* strains harboring these sulbactam resistance mutations may potentially demonstrate decreased virulence in patients [6].

The frequency of spontaneous resistance to sulbactam–durlobactam in four clinical isolates ranged from 7.6 × 10^−10^ to < 9.0 × 10^−10^ at 4 × MIC, and this resistance was associated with residues near the active site of PBP3. Stable mutant isolates demonstrated MICs 8 to >32 times the MIC of the parental strain. It is worth noting that S390T and S395F were created in the lab and were not found in clinical isolates [25].

### 2.3. Pharmacokinetics and Pharmacodynamics

The pharmacokinetic properties of sulbactam–durlobactam are well studied. When administered intravenously, both sulbactam and durlobactam are rapidly distributed in the bloodstream. They exhibit relatively short half-lives, necessitating multiple doses for optimal therapeutic effect. Dosing regimens may need to be designed according to the specific location of infection and individual patient characteristics. Pharmacodynamic studies have shown that ensuring the presence of adequate drug concentrations exceeding the MIC is pivotal for achieving effectiveness, underscoring the significance of optimizing the dosage. The individual pharmacokinetic parameters of sulbactam and durlobactam are mentioned in Table 2.

The pharmacokinetics of both sulbactam and durlobactam are similar when administered as single doses or multiple doses [26]. The *C*_max_ and AUC of sulbactam increase proportionally with the administered dosage [15]. Durlobactam demonstrated a dose-dependent pharmacokinetics among the studied range of doses [26].

Like other *β*-lactam-*β*-lactamase inhibitors, sulbactam–durlobactam is mainly eliminated from the body through renal excretion. Around 75% of both components are excreted unchanged in the urine, and the remaining 25% are metabolized through noncytochrome P450-mediated hydrolytic cleavage and subsequent clearance. Renal impairment significantly affects the pharmacokinetics of sulbactam–durlobactam. A phase 1 study evaluated the impact of different levels of renal dysfunction, including individuals with end-stage renal disease (ESRD) on hemodialysis (HD), on the pharmacokinetic parameters and side effects of sulbactam and durlobactam following a single IV dose administration. For healthy patients and those with mild renal dysfunction (creatinine clearance (CrCl) between 60 and 90 mL/min)) or moderate renal dysfunction (CrCl between 30 and 60 mL/min), a single 1 g dose of each sulbactam and durlobactam was administered over a 3-h infusion. For patients with severe renal dysfunction (CrCl less than 30 mL/min), 0.5 g doses were given. For patients with ESRD and HD, 0.5 g doses each of durlobactam and sulbactam were given pre-HD and post-HD. There was a 1-week washout period between the doses. Pharmacokinetic parameters for sulbactam and durlobactam were similar across most concentrations of each agent. As the renal function decreased, there was a linear increase in blood concentrations for all participants, irrespective of their baseline kidney function. In healthy individuals and in those with mild or moderate renal dysfunction, the majority of sulbactam and durlobactam was excreted in the urine, while in subjects with severe renal dysfunction or ESRD, approximately up to 40% of the drug was eliminated in the urine. Both sulbactam and durlobactam were removed considerably by HD. Renal impairment did not affect the safety and tolerability of either sulbactam or durlobactam. Nevertheless, the prescribed dose of sulbactam–durlobactam will need reduction in patients with severe renal dysfunction and ESRD [26].

Trial data also concluded that when sulbactam and durlobactam are coadministered, there is no substantial change in the pharmacokinetics of either agent. Another phase I study analyzed the pharmacokinetic parameters and subsequent concentrations of sulbactam–durlobactam in the epithelial lining fluid (ELF) and alveolar macrophage (AM) of 30 healthy individuals. Participants were given 1 g of each sulbactam and durlobactam over 3-h infusions every 6 hours for three successive doses. When analyzing the distribution of durlobactam in the ELF, the AUC concentration over a 6-h period was found to be 40.1 h mg/L. The ratio of ELF to total plasma was 0.36. These findings regarding intrapulmonary concentrations show that sulbactam–durlobactam is a promising option for the treatment of respiratory infections [28].

In vitro models of infection revealed that the most accurate predictor of effectiveness for sulbactam is the percentage of time within the dosing interval during which unbound plasma levels of sulbactam surpass the MIC needed to inhibit the growth of *A. baumannii* (*f*T > MIC 50%). To evaluate the efficacy of exposure, a pharmacokinetic and pharmacodynamic (PK/PD) analysis of sulbactam was conducted utilizing murine thigh and lung infection models of *A. baumannii* [29].

The PK/PD index that most closely correlated with its in vivo effects was the duration for which the free drug concentration remained above the minimum inhibitory concentration.

This study showed that sulbactam was effectively bactericidal when the fT > MIC exceeded 60% for *A. baumannii* thigh infections and 40% for *A. baumannii* lung infections. In vivo and in vitro infection models showed that the most accurate predictor of effectiveness for durlobactam is the ratio of the 24-h unbound plasma durlobactam AUC to the sulbactam–durlobactam MIC (*f*AUC_0–24_/MIC ≥ 10) [15]. For sulbactam–durlobactam combination, an in vitro infection model against a single multidrug-resistant *A. baumannii* strain showed that fT > a CT of 0.75 mg/L was strongly associated with the drug's efficacy. Because of durlobactam's relatively short half-life, it was shown that the fAUC_0–24_ served as a more accurate predictor of effectiveness as compared to fT > CT [30].

In a study focused on multidrug-resistant *Acinetobacter* species, the addition of durlobactam significantly enhanced the susceptibility of clinical *A. baumannii* isolates to sulbactam. The in vivo effectiveness of sulbactam–durlobactam was compared to colistin in a murine thigh infection model. This evaluation was conducted using a multidrug-resistant strain of *A. baumannii* (ARC5955). Sulbactam exhibited an MIC of 64 μg/mL when used alone. When combined with durlobactam, the MIC significantly decreased to 4 μg/mL [31].

### 2.4. Efficacy

Kaye et al. evaluated the efficacy and safety of sulbactam–durlobactam versus colistin in patients with serious infections caused by CRAB in the ATTACK trial, a phase 3, pathogen-specific, multinational, randomized, active-controlled, noninferiority study [32]. Patients with confirmed ABC HABP, VABP, ventilated pneumonia, or bloodstream infections were equally randomized to receive sulbactam–durlobactam (1 g of each drug administered via an IV infusion over 3 h every 6 h) or colistin (a loading dose of 2.5–5.0 mg/kg (75,000–150,000 IU/kg), followed by a maintenance dose of 2.5 mg/kg (75,000 IU/kg) via IV infusion over 30 min every 12 h). Patients were additionally administered imipenem–cilastatin to cover possible coinfecting pathogens. Study participants were stratified according to infection type, disease severity, and geographic region. Patients with a known history of allergy to any *β*-lactam, or any contraindication to the use of imipenem–cilastatin or pulmonary disease that could confound the evaluation of efficacy, were excluded from the study. The duration of therapy was 7–14 days. The primary endpoint was 28-day all-cause mortality in the CRAB microbiologically modified intention-to-treat (MITT) population defined as patients with a baseline ABC organism confirmed to be carbapenem-resistant, assessed using a noninferiority margin of 20%. Secondary endpoints comprised 28-day all-cause mortality in the ITT, microbiologically modified ITT, and CRAB microbiologically evaluable populations and 14-day all-cause mortality in the CRAB microbiologically modified ITT and microbiologically modified ITT populations in addition to clinical cure and microbiological favorable assessment at the end of treatment, test of cure, and late follow-up. For safety, treatment-emergent adverse events (TEAEs), serious AEs, and nephrotoxicity were monitored as outcomes. One hundred eighty-one patients were enrolled from 59 clinical sites in 16 countries, among which 128 comprised the CRAB microbiologically modified ITT population. The CRAB microbiologically modified ITT population featured a male predominance (74%) enrolled primarily from Europe (41%) and China (27%). Their demographic profile revealed a median age of 63 years, while their clinical condition at baseline was characterized by an average APACHE II score of 16.8. It is noteworthy that a substantial portion, specifically 40% of this cohort, exhibited concurrent renal impairment at baseline. The majority of patients (97.6%) in the primary endpoint population had pneumonia as a primary source of infection (55 patients with HAP, 68 VAP, and two ventilated pneumonia), and three had bacteremia. Patients' characteristics across the treatment groups were largely homogenous, with minor differences observed. Particularly, a higher proportion of patients in the colistin group were 75 years of age (33%) compared to the sulbactam–durlobactam group (19%). Additionally, there was a higher enrollment of Asian patients in the colistin group (53%) than in the novel drug group (36%). Furthermore, the sulbactam–durlobactam group exhibited a higher percentage of patients with polymicrobial infection at baseline (42%) compared to the colistin group (30%).  Table 3 summarizes the efficacy outcomes. A second component of the ATTACK trial investigates in an open-label study the efficacy of sulbactam–durlobactam in treating ABC-infected patients who were resistant to colistin or polymyxin B, as well as those who had failed colistin or polymyxin B regimen before entering the study. A total of 28 patients were enrolled in this group among which 61% had bloodstream infections and 64% had an ICU stay of more than 14 days. The 28-day all-cause mortality rate was 18% (95% CI 6–37). The results demonstrate the efficacy of sulbactam–durlobactam in patients with colistin-resistant infections including bloodstream infections.

The ATTACK trial's findings support sulbactam–durlobactam as a favorable treatment option for serious infections caused by CRAB, including multidrug-resistant strains. Moreover, the novel drug showed promising results for the treatment of colistin-resistant infections, albeit warranting further rigorous investigation and scrutiny.

Nevertheless, limitations of the ATTACK trial shall not be ignored. Indeed, severely ill patients with an APACHE score > 30 and a SOFA score > 11 or receiving peritoneal dialysis were excluded. The underrepresentation of females as well as the North American and Western European populations limits the generalizability of the trial to these areas of the world [32]. While the results tentatively endorse the efficacy of sulbactam–durlobactam in patients with bloodstream infections, it is imperative to note that the findings lack robustness in this group of people due to the limited number of enrolled patients with bloodstream infections in the study.

### 2.5. Safety and Tolerability

Durlobactam, with or without sulbactam, was evaluated for safety and tolerability in 380 adult subjects across six phase 1 trials and one phase 2 trial in patients with complicated urinary tract infections (cUTIs) including acute pyelonephritis [30], and in the ATTACK trial [31]. The phase II trial is a double-blind, randomized, and placebo-controlled. Patients were randomly assigned to a 2:1 ratio to receive either sulbactam–durlobactam (1 g of each drug intravenously every 6 h) or placebo, both in addition to imipenem–cilastatin (500 mg intravenously every 6 h) as background therapy for a total of 7 days. Patients diagnosed with bacteremia received therapy for up to 14 days. This study assessed the tolerability and pharmacokinetics of sulbactam–durlobactam in patients with cUTIs. This focus is particularly important given that ABC is linked to serious infections, including cUTIs. The ATTACK phase III trial studied the safety and efficacy of sulbactam–durlobactam in HABP and VABP ABC infections [27, 32]. When comparing arms in terms of tolerability, reported treatment-related AEs were mostly mild or moderate in severity without any serious AEs [27]. Both groups had comparable incidence of AEs: 12 (22.6%) patients treated with sulbactam–durlobactam and 4 (14.8%) patients treated with placebo. Treatment-related AEs included headache (5.7%), nausea (3.8%), diarrhea (3.8%), and vascular pain (3.8%) in the sulbactam–durlobactam group. Two patients discontinued treatment; one patient treatment was stopped due to urticaria and the other was stopped due to an increase in serum creatinine and the protocol didn't allow for an imipenem dose reduction. No death was reported [27]. In the ATTACK trial, sulbactam–durlobactam was well tolerated. Lower incidence of drug-related AEs was recorded and the incidence of nephrotoxicity was lower in the sulbactam–durlobactam compared to the colistin group (12 [13%] of 91 vs. 32 [38%] of 85, (*p* < 0.001)) [33]. Common drug-related AEs in the sulbactam–durlobactam with more than 10% occurrence rate are increase in liver test abnormalities (19 [17%]), diarrhea (17[15%]), anemia (13[12%]), and hypokalemia (12[11%])). Neutropenia also occurred in one patient on sulbactam–durlobactam and it resolved on day 12 of treatment. Emergent infections or superinfections and *C. difficile* infections occurred in both arms of the study; however, they were more frequent with colistin (25 [29%] of 86 and 6 [7%] of 86) than with sulbactam–durlobactam (17 [19%] of 91] and 1 [1%] of 91, respectively. Serious AEs were experienced in both groups: 36 patients (40%) in the Xacduro treatment group and 42 patients (49%) in the colistin treatment group. Treatment-related AEs leading to study drug discontinuation were also reported in 10 (11%) of 91 patients in the sulbactam–durlobactam group and 14 (16%) of 86 patients in the colistin group. In addition, an incidence of anaphylactic shock due to sulbactam–durlobactam was reported and led to discontinuation of treatment and the initiation of supportive management [32].  Table 4 summarizes the most common AEs in the clinical trials.

### 2.6. Drug Interactions

Sulbactam–durlobactam appears to have a low drug–drug interactions risk based on the pharmacokinetic studies [33]. However, sulbactam and durlobactam are both substrates of organic anion transporter 1 (OAT1). Only sulbactam is predicted to have active secretion as a significant portion of total clearance. Sulbactam plasma concentration is increased with concomitant administration of OAT1 inhibitors (such as probenecid); therefore, coadministration is not recommended although there are no clinical studies conducted with sulbactam–durlobactam and OAT1 inhibitors.

In vitro studies showed no antagonism between sulbactam–durlobactam and other antibiotics [15, 34].

## 3. Special Population [15, 34]

### 3.1. Pregnancy

For the combination of sulbactam durlobactam, there are no available data on its use in pregnancy to evaluate for a drug-related risk of major birth defects, miscarriage, or other adverse maternal or fetal outcomes. Data is available on individual components of Xacduro. Individually, both medications, sulbactam and durlobactam, are not reported to have any drug-related risk of major birth defects, miscarriage, or other adverse maternal or fetal outcomes when used in combination with ampicillin during pregnancy.

### 3.2. Lactation

Sulbactam has been reported to be present in human milk in low concentrations, while no data is available on the presence of durlobactam in human or animal milk. To date, there is no information on the effects of Xacduro, sulbactam, or durlobactam on the milk production or the breastfed infant.

### 3.3. Pediatrics

The safety and effectiveness of Xacduro have not been established in patients younger than the age of 18 years.

### 3.4. Geriatrics

Clinical studies of Xacduro did not include adequate numbers of patients aged 65 and over to assess whether there is difference in response to treatment compared to younger patients.

### 3.5. Dosing in Renal Impairment

Xacduro does not require dosage adjustment in patients with CrCl 45–129 mL/min. Dose adjustment is recommended for patients with CrCl less than 45 mL/min including patients receiving intermittent HD. In patients requiring HD, administer Xacduro dose right before ending HD. Limited information is available for patients receiving Continuous Renal Replacement Therapy (CRRT); therapy should be directed by the patient's clinical status. While on CRRT, a patient's residual renal function may change, which may warrant a change in Xacduro dosage. Seriously ill patients who are receiving intravenous fluid resuscitation may have a CrCl of 130 mL/min or greater with an increase in the clearance of the medication and will require the medication to be given more frequently.

### 3.6. Dosing in Hepatic Impairment

Dosage adjustments are not necessary in patients with impaired hepatic function since none of the components of Xacduro undergo substantial hepatic metabolism or excretion. To add, the effects of hepatic impairment on the pharmacokinetics of sulbactam and durlobactam have not been evaluated.

### 3.7. Dosing, Administration and Cost

The recommended dose of sulbactam–durlobactam in adults with CrCl between 45 mL/min and 130 mL/min is 1 g (g) of sulbactam and 1 g of durlobactam every 6 h administered by intravenous (IV) infusion over 3 h [15].The duration of treatment is directed by the patient clinical response, and it ranged between 7 and 14 days. The dosage should be adjusted in patients with renal impairment as defined by a CrCl of less than 45 mL/min and in patients with CrCl greater than 130 mL/min.  Table 5 specifies the dosing recommendations based on CrCl [35]. When first approved, Xacduro was supplied as a co-packaged kit containing 1 clear single-dose vial of sulbactam 1 g and 2 amber single-dose vials of durlobactam 0.5 g as sterile powders. However, in October 2023, in response to the Centers for Disease Control and Prevention (CDC) National Healthcare Safety Network (NHSN) feedback regarding issues with antimicrobial use surveillance and the modeling of Xacduro in RxNorm, Xacduro formulation was changed to include 1 g of sulbactam and 1 g of durlobactam [35, 36]. The sterile powders must be reconstituted and further diluted prior to intravenous infusion. Since Xacduro does not contain a bacteriostatic preservative, once mixed, it must be used within 24 h when stored refrigerated at 2°C–8°C (36 °F–46 °F). Discard unused portion. To note, the prepared solution must be placed at room temperature for 15–30 min prior to infusion. Xacduro is compatible with 0.9% sodium chloride injection, USP. To prepare a dose of Xacduro, reconstitute the sulbactam 1-g single-dose vial with 5 mL of sterile water for injection and gently shake to dissolve. Reconstitute durlobactam 1-g single-dose vial with 5 mL of sterile water for injection and gently shake to dissolve. Additional dilution of both solutions in 0.9% sodium chloride must occur within 1 h of reconstitution. In order to prepare the required Xacduro dose, we withdraw 5 mL of reconstituted sulbactam and 5 mL of reconstituted durlobactam and then add the withdrawn volume of both sulbactam and durlobactam to a 100 mL infusion bag of 0.9% sodium chloride for injection, USP. We discard unused portion. We do not administer or mix Xacduro with solutions containing other drugs or other diluents since compatibility has not been established [15, 34, 37].

The average wholesale price (AWP) of one day of treatment with sulbactam–durlobactam is $760, which is less expensive when compared to other new beta-lactam beta-lactamase inhibitors (the AWP of one day of treatment with imipenem–relebactam is $1430, and that with ceftazidime–avibactam is $1356 [36, 38, 39]. However, pharmacoeconomic studies on the use of sulbactam–durlobactam have not been published yet.

### 3.8. Place in Therapy

Sulbactam–durlobactam is restricted to be only used in the treatment of HABP/VABP caused by susceptible isolates of ABC despite the fact that its tolerability and pharmacokinetics were studied in patients with cUTI and bacteremia. Sulbactam–durlobactam was tolerated with AEs classified as mild to moderate with no severe reactions. Therefore, a possible potential for its use would be in the case of complicated UTI and bacteremia. In addition, the use of sulbactam–durlobactam combined with cefiderocol appears promising for the treatment of extensively resistant *A. baumannii* in patients with pneumonia and septic shock based on a case report where a patient with *A. baumannii* pneumonia and septic shock was successfully treated [40]. Only one phase III trial studied the safety and efficacy of sulbactam–durlobactam HABP/VABP caused by susceptible isolation of ABC complex. Xacduro showed noninferiority when compared to colistin, and both groups also received imipenem–cilastatin. According to the IDSA 2024 Guidance on the treatment of antimicrobial-resistant Gram-negative infections, a sulbactam-containing regimen is recommended for CRAB management, with sulbactam–durlobactam being the preferred option when combined with a carbapenem since sulbactam–durlobactam is the first antibiotic to demonstrate efficacy in a 28-day all-cause mortality trial focused on CRAB. In cases where sulbactam–durlobactam is unavailable, alternatives such as high-dose ampicillin–sulbactam with other agents (e.g., polymyxins, minocycline, or cefiderocol) are suggested. Head-to-head clinical trials comparing sulbactam–durlobactam with other common regimens are needed in the future [41]. In terms of safety, sulbactam–durlobactam significantly resulted in less nephrotoxicity and treatment-related AEs. Additionally, sulbactam–durlobactam has a potential role in treating polymyxins-resistant ABC and colistin or polymyxin regimen failure as demonstrated as part of the ATTACK trial. The results of the ATTACK trial support its efficacy in colistin-resistant infections including bloodstream infections [33].

Accordingly, the use of sulbactam–durlobactam should be reserved for the treatment of infections caused by ABC infections that are resistant to other available agents for the treatment of HABP and VABP although sulbactam–durlobactam should be considered before the use of polymyxins for the treatment of ABC, particularly in patients at high risk of nephrotoxicity. Clinical trials, the development of other new agents, are active against resistant Gram-negative organisms particularly ABC, and pharmacoeconomic studies will determine the specific role of sulbactam–durlobactam in the treatment of resistant Gram-negative infections.

## 4. Conclusion

Sulbactam–durlobactam, a new *β*-lactam-*β*-lactamase inhibitors combination, well tolerated with a favorable safety profile, is indicated in patients 18 years of age and older for the treatment of HABP/VABP caused by susceptible isolates ABC that are resistant to carbapenems.

## Figures and Tables

**Table 1:** MIC 50/90 values of sulbactam–durlobactam for *Acinetobacter* baumannii complex isolates [19].

Organism	Number of isolates	Antibiotic	MIC50 (mg/L)	MIC90 (mg/L)
ABC	5032	Colistin	0.5	1
		Imipenem	8	> 64
		Sulbactam–durlobactam	1	2
		Sulbactam	8	64
		Tigecycline	0.5	2

*Acinetobacter baumannii*	982	Colistin	0.5	1
		Imipenem	> 8	> 8
		Sulbactam–durlobactam	1	4
		Sulbactam	32	> 64
		Tigecycline	2	4

Carbapenem-resistant *Acinetobacter baumannii*	246	Colistin	0.5	1
		Sulbactam–durlobactam	1	4
		Sulbactam	16	64
		Minocycline	2	16
		Amikacin	256	> 512

Carbapenem-resistant *Acinetobacter baumannii*	190	Colistin	2	16
		Sulbactam–durlobactam	4	8
		Sulbactam	64	> 64
		Minocycline	16	32
		Amikacin	> 128	> 128

Abbreviations: ABC, *Acinetobacter baumannii*–calcoaceticus complex; MIC, minimum inhibitory concentration.

**Table 2:** Pharmacokinetic parameters (mean ± SD) of sulbactam and durlobactam [26, 28].

Pharmacokinetic parameter	Sulbactam	Durlobactam
*C*_max_ (mg/L)	32.4 ± 24.7	29.2 ± 13.2
*C*_min_ (mg/L)	5.79 ± 2.08	2.50 ± 1.09
AUC_0-24_ (h·mg/L)	515 ± 458	471 ± 240
% Plasma protein binding	38%	10%
Volume of distribution at steady state (L)	25.4 ± 11.3	30.3 ± 12.9
Clearance (L/h)	11.6 ± 5.64	9.96 ± 3.11
Half-life (h)	2.15 ± 1.16	2.52 ± 0.77
Main route of elimination	Renal	Renal
% Excreted unchanged in urine	75%–85%	78%

Abbreviations: AUC_0–24_ = area under the plasma concentration–time curve from time of dosing to 24 h; *C*_max_ = maximum concentration; *C*_min_ = minimum concentration; SD = standard deviation.

**Table 3:** Efficacy outcomes in the ATTACK trial [32].

	Sulbactam–durlobactam % (n = 63)	Colistin % (*n* = 62)	Treatment difference, % (95% CI)
*Primary endpoint*			
28-Day all-cause mortality in carbapenem-resistant ABC microbiologically modified ITT	19.0	32.3	−13.2 (−30.0 to 3.5)

*Secondary endpoints*			
28-Day all-cause mortality in the ITT	17	36	−19 (−39.1 to 1.2)
28-Day all-cause mortality in the microbiologically modified ITT	20	33	−13 (−28.3 to 2.0)
28-Day all-cause mortality in the carbapenem-resistant ABC microbiologically evaluable populations	21	33	−12 (−26.0 to 2.4)
14-Day all-cause mortality in the carbapenem-resistant ABC microbiologically modified ITT	6	19	−13 (−25.7 to 0.1)
14-Day all-cause mortality in the microbiologically modified ITT	8	20	−12 (−23.7 to 0.3)

*Clinical cure*			
End of treatment	75	45	29% (11.4–47.4)
Test of cure	62	40	22% (2.9–40.3)
Late follow-up	43	31	12% (−6.2–30.6)

*Microbiological favorable assessment*			
Response at end of treatment	86	61	24% (7.9–40.9)
Response at test of cure	68	42	26% (7.9–44.7)
Sustained response at late follow-up	48	40	7% (−11.7–26.3)
Persistence at late follow-up	27	52	
Recurrence at late follow-up	10	3	
Indeterminate at late follow-up	16	5	

Abbreviations: ABC, *Acinetobacter baumannii*–calcoaceticus complex; CI, confidence interval; ITT, intention-to-treat.

**Table 4:** Common treatment-related adverse events (AEs) observed in clinical trials sulbactam–durlobactam [28, 32].

Clinical trial	Treatment-related AE	Incidence (%)
Phase II trial: pharmacokinetics and tolerability of intravenous sulbactam–durlobactam with imipenem–cilastatin in hospitalized adults with complicated urinary tract infections, including acute pyelonephritis	Headache	5.7
	Nausea	3.8
	Diarrhea	3.8
	Vascular pain	3.8
	Urticaria	1.9
	Nephrotoxicity	1.9

Phase III trial: efficacy and safety of sulbactam–durlobactam versus colistin for the treatment of patients with serious infections caused by acinetobacter baumannii–calcoaceticus complex: a multicenter, randomized, active-controlled, phase 3, noninferiority clinical trial (ATTACK)	Liver test abnormalities	17
	Diarrhea	15
	Anemia	12
	Hypokalemia	11

**Table 5:** Dosage and administration of Xacduro (SUL-DUR, sulbactam, and durlobactam) for patients (18 years of age and older) according to CrCl [15, 34].

Dose of sulbactam–durlobactam	Frequency	CrCl (mL/min)
Sulbactam 1 g and durlobactam 1 g	Q 4 h	Greater than 130
	Q6 hours	45–130
	Q8 hours	30–44
	Q 12 h	15–29
	Patients already receiving SUL-DUR whose CrCl declines to less than 15 mL/min: administer every 24 h	Less than 15^∗^
	Patients to be initiated on SUL-DUR: administer the first 3 doses every 12 h (0, 12, and 24 h) and then maintain a regimen of every 24 h	

^∗^Administer at the end of hemodialysis for patients on HD.

## Data Availability

The data that support the findings of this study are available on request from the corresponding author.
